# Medical decisions concerning the end of life for cancer patients in three Colombian hospitals – a survey study

**DOI:** 10.1186/s12904-021-00853-9

**Published:** 2021-10-18

**Authors:** Esther de Vries, Fabián Alexander Leal Arenas, Agnes van der Heide, Fritz E. Gempeler Rueda, Raul Murillo, Olga Morales, Eduardo Diaz-Amado, Nelcy Rodríguez, Beatriz Juliana Gonzalez, Danny Steven Castilblanco Delgado, Jose A. Calvache

**Affiliations:** 1 Department of Clinical Epidemiology and Biostatistics, Pontificia Universidad Javeriana, Cra. 7 No 40-62 Edificio Hospital San Ignacio, Piso 2, Bogota, Colombia; 2 Instituto Nacional de Cancerología, Cl. 1 No 9-85, Bogota, Colombia; 3 Centro Javeriano de Oncología, Hospital Universitario San Ignacio, Carrera 7ª No. 40-62 Edificio Santacoloma (No 30), Bogota, Colombia; 4 Department of Public Health, Erasmus MC University Medical Center Rotterdam, PO Box 2040, 3000 CA Rotterdam, The Netherlands; 5 Anesthesiology Department, Pontificia Universidad Javeriana, Cra. 7 No 40-62 Edificio Hospital San Ignacio, Bogota, Colombia; 6 Clinical Ethics Service, Hospital Universitario San Ignacio, Cra. 7 No 40-62, Bogota, Colombia; 7 Department of Internal Medicine, Pontificia Universidad Javeriana, Cra. 7 No 40-62 Edificio Hospital San Ignacio, Bogotá, Colombia; 8 Instituto de Bioética, Pontificia Universidad Javeriana, Tv. 4 #42, Bogota, Colombia; 9 Universidad Militar Nueva Granada, Carrera 11 n.° 101-80, Bogota, Colombia; 10 Department of Anesthesiology, Universidad del Cauca, Cl 5 #4-70, Popayán, Cauca Colombia; 11 Department of Anesthesiology, Erasmus MC University Medical Center Rotterdam, PO Box 2040, 3000 CA Rotterdam, The Netherlands

**Keywords:** End of life, Palliative care, Decision making, Medical decision, Colombia, Cancer, Euthanasia, Palliative sedation

## Abstract

**Background:**

Cancer patients’ end-of-life care may involve complex decision-making processes. Colombia has legislation regarding provision of and access to palliative care and is the only Latin American country with regulation regarding euthanasia. We describe medical end-of-life decision-making practices among cancer patients in three Colombian hospitals.

**Methods:**

Cancer patients who were at the end-of-life and attended in participating hospitals were identified. When these patients deceased, their attending physician was invited to participate. Attending physicians of 261 cancer patients (out of 348 identified) accepted the invitation and answered a questionnaire regarding end-of-life decisions: a.) decisions regarding the withdrawal or withholding of potentially life-prolonging medical treatments, b.) intensifying measures to alleviate pain or other symptoms with hastening of death as a potential side effect, and c.) the administration, supply or prescription of drugs with an explicit intention to hasten death. For each question addressing the first two decision types, we asked if the decision was fully or partially made with the intention or consideration that it may hasten the patient’s death.

**Results:**

Decisions to withdraw potentially life-prolonging treatment were made for 112 (43%) patients, 16 of them (14%) with an intention to hasten death. For 198 patients (76%) there had been some decision to not initiate potentially life-prolonging treatment. Twenty-three percent of patients received palliative sedation, 97% of all patients received opioids.

Six patients (2%) explicitly requested to actively hasten their death, for two of them their wish was fulfilled. In another six patients, medications were used with the explicit intention to hasten death without their explicit request. In 44% (*n* = 114) of all cases, physicians did not know if their patient had any advance care directives, 26% (*n* = 38) of physicians had spoken to the patient regarding the possibility of certain treatment decisions to hasten death where this applied.

**Conclusions:**

Decisions concerning the end of life were common for patients with cancer in three Colombian hospitals, including euthanasia and palliative sedation. Physicians and patients often fail to communicate about advance care directives and potentially life-shortening effects of treatment decisions. Specific end-of-life procedures, patients’ wishes, and availability of palliative care should be further investigated.

**Supplementary Information:**

The online version contains supplementary material available at 10.1186/s12904-021-00853-9.

## Background

When cure is no longer the main treatment objective for patients with cancer, physicians, patients and family often are faced with making difficult decisions; for example, whether or not to use potentially life-prolonging medical treatment that is burdensome, risky or with very low probability of success. Decisions should also involve questions on the desirability, effectiveness and safety of extensive diagnostic procedures, surgeries, hospitalization or admission to intensive care units [1]. When cancer patients enter into the last phase of their lives, they often suffer substantially due to pain or other symptoms, or even from the absence of any perspective on improvement. In such situations, decisions need to be made regarding patient comfort [1]. These decisions may affect the remaining duration of the patient’s life, their subjective experience during that time or help to address their wishes at the time of death and beyond. Some patients experience their suffering in the last phase of life as unbearable and ask their doctor for euthanasia. Euthanasia is defined as follows: a physician intentionally ending a person’s life by the administration of drugs, at that competent person’s voluntary request [2].

The relative frequency of these decisions varies between countries. Particularly when decisions which actively hasten the end of life are concerned, public and professional debates are extensive. Information regarding the relative frequencies and circumstances of these decisions can help nurture a fruitful debate [3–7].

Colombia, a middle-income country in South America, exhibits an increasingly older population due to a transition from a mortality pattern dominated by unnatural causes and communicable diseases towards one dominated by chronic diseases [8]. This growing number of patients dying from chronic diseases has led to the introduction of palliative care in the country, mainly concentrated in hospitals, but slowly spreading towards home care as well [9–11]. Since 2014, Colombia has a law on palliative care, which regulates what palliative care is, who should have access to palliative care and under which circumstances [11, 12]. Consciousness in society and the medical community at large that end-of-life care for patients with chronic diseases may involve difficult decision-making processes, is slowly growing.

Colombia is the only country in South America that has adopted regulation regarding euthanasia (see Table 1) [13] – the number of registered euthanasia procedures has risen from 5 in 2015 to 44 in 2019, a figure which dropped to 26 in “covid-19” year 2020 [14] . These figures only represent those reported to the ministry and which therefore complied with all regulations. It is impossible to know how many “euthanasias” were performed. The current regulation has been heavily debated; the offer of palliative care in the country is still very deficient and the critics argue that palliative care should be offered to avoid euthanasia requests [9, 10]. A small study showed lack of knowledge among university students regarding euthanasia, but also quite a high degree of acceptance [15]. High quality home- and institution-based palliative care that is available to all is considered paramount in order to ensure that people don’t request euthanasia because of insufficient symptom control, lack of access to care or fear of being a burden to caregivers [16].

**Table 1:** The legal status of the application of euthanasia in Colombia in 2017–2020

In 1997, the Constitutional Court stated the conditions for practicing euthanasia (defined as “murder out of mercy” in the Penal Code) and ruled that under such conditions there would not be any penalty. This was a paradoxical outcome as the Court was solving a case in which a Colombian citizen was requesting to increase the penalty for “killing out of mercy” (six months to three years) to be comparable to homicide (10+ years). In the case of euthanasia, the Court acknowledged that the active subject (the doctor) was acting within the criterion of compassion and solidarity, which is enshrined in the Constitution, for the passive subject (the patient). The Court considered that euthanasia must be requested by the patient himself, who must suffer from a terminal illness that causes intense suffering and which cannot be otherwise alleviated. It was established that euthanasia must be carried out by a physician, who would not be penalized if conditions for euthanasia were met (see below). The treating physician must know the clinical condition of the patient to such extent that a good prediction of prognosis (using prediction scales) is possible in order to define if the patient can be considered terminally ill. A terminally ill patient is defined as a patient with a medically confirmed advanced, progressive and uncontrollable disease, characterized by the absence of reasonable treatment options, with physical and psychological suffering despite having received the best available treatment, and with a life expectancy of less than 6 months. The following are the processes required to guarantee that patient is capable of requesting euthanasia, regulated in the Protocol for the application of the procedure of Euthanasia in Colombia [11], 1: Medical condition: terminally ill patient. Expected date of death in absence of euthanasia must be established, communicated to the patient and registered in the medical record. Physician must also record if patient is considered to understand his or her medical condition. 2: Evaluation of suffering: nature and level of suffering must be evaluated as intolerable and without perspectives of improvement. This evaluation must include the perception of the treating physician and the patients´ perception, prioritizing the latter. 3: Absence of alternative treatment or care options. Received interventions must be documented, including those related to symptom management and palliative care and the results of these interventions. The patient must have had contact with a specialist in pain and palliative care and a disease-specific specialist. 4: Persistence of the explicit request. Treating physician informs when was the first time the patient expressed the request and if this request persisted over time for at least 25 days or was repeated and if the request is voluntary, free of influence of others or if any “advance directive”, either written or documented in the medical file, exists. 5: Evaluation of the capacity to decide. A psychiatrist or clinical psychologist must establish the capacity to make decisions of the patient. This evaluation must be performed prior to the evaluation by the euthanasia committee. 6: Second evaluation. The scientific interdisciplinary committee for the right to a dignified death, is the evaluates if the anterior requisites are fulfilled. This committee must be independent from the treating physician (in particular in terms of hierarchy), must not have evaluated the patient previously, and must not have a personal or professional relation with the patient. In case of discordance between the two evaluations, the committee re-evaluates the case consulting another professional. 7: Integrity of the evaluation. The treating physician and the scientific interdisciplinary committee for the right to a dignified death must base their evaluation on the medical records, the document with the written request, the conversation with and physical examination of the patient, and the dialogue with other members of the team of physicians or the family, if the patient authorizes. The treating physician must provide a summary of these findings at the moment of presenting the request for the committee. Patients and family members or caretakers must be informed on each step of the process and be accompanied by psychologists if needed. Physicians or medical institutions can refuse to provide euthanasia to a terminally ill patient: physicians can take on conscientious objection and institutions can refuse arguing that their principles are violated, but they have to guide the patient towards a place or situation where their request can be met. Both adults and children (from 6 years on, meeting certain conditions) are legally allowed to request the procedure. It is compulsory to send a complete report of the case to the Ministry of Health and Social Protection, which will check the standards of the procedure and provide statistics about it.	

The objective of this study was to document current practices of medical decisions concerning the end of life, that is, decisions to refrain from potentially life-prolonging treatment and decisions to use (potentially) life-shortening medication of cancer patients in Colombia. This information can serve as a base to nurture future discussion regarding rules and regulations and the public debate in the precarious field of end-of-life decisions and thereby can help guide health care professionals, policy-making and the public in general.

## Methods

### Study design and setting

This cross-sectional survey study was exploratory and descriptive in nature. We documented medical decisions taken during the last month of life of 261 cancer patients who died between May 2019 and May 2020 in one of three teaching hospitals: Instituto Nacional de Cancerología (INC), Hospital Universitario San Ignacio (HUSI) and Hospital San José (HUSJ). The first two are located in Bogota, the INC being a specialized and public cancer referral hospital, attending over 7000 new patients per year, and HUSI being a non-profit tertiary hospital – all three hospitals have specialized oncology centers or departments and palliative care teams. HUSJ is a public hospital in a Colombian province, in the city of Popayán, attending the urban population (> 300,000 inhabitants) and a large rural area, including several indigenous tribes (guambianos and paéces). Colombia has a mandatory “universal” national social insurance scheme including two main insurance schemes, a contributory one financed by payroll contributions and a subsidized scheme for the poorest population by general taxation. In addition, there are special and exceptional groups which consist of specific types of government workers (public teachers, military, police and state oil company) with their own schemes [17]. The three participating hospitals attend patients affiliated with different schemes: HUSI mostly attends patients covered under the contributory scheme, while INC and HUSJ attend patients under both schemes as well as patients from special and exceptional schemes.

### Participant selection

Beginning in May 2019 at HUSI, any patient with cancer in final stages and a life expectancy of about 3 months or less who was seen in any of the three clinics was eligible for the study. These patients were identified by nurses and physicians in participating hospitals who were asked to notify the research team when any patient with these characteristics was seen at the outpatient clinic, emergency department or inpatient wards, without specifying particularly which criteria to use the life expectancy (in practice, physicians use different functional scales and progressive deterioration of the patients to assess this life expectancy). The only inclusion criteria were for patients to have cancer, a life expectancy of 3 months or less (as evaluated by the hospital team – no explicit instructions) and being attended at least once in one of the participating hospitals. There were no exclusion criteria. Research assistants followed-up these patients every week in hospital and governmental systems to assess whether they were still alive or not and to assess date of death for the deceased patients. When these patients deceased (either in hospital or at home), a research assistant assigned a case number to the patient, obtained basic information such as sex, age, type of cancer and type of health insurance (contributive or subsidized, special/exceptional [17]) from the medical file and invited one of the attending physicians to fill out a questionnaire. This physician was selected based on involvement with the patient’s care at the end-of-life. The physician was asked to forward the questionnaire to a colleague if he felt the colleague had a better understanding of the decisions surrounding the patient. As a result of this process, it is possible that some physicians answered the questionnaire for more than one patient. When within about 5 days of inviting the physician no reply was obtained on the questionnaire, the research assistant contacted the physician as a reminder.

The sample of this study cannot be considered as randomized – a randomized sampling strategy was impossible because of the absence of a sampling frame in Colombia. There is a death certificate registration (vital statistics) but privacy laws make it impossible for researchers to know the identifying information of the deceased and additionally it would be impossible to find out who treated the patients. The study therefore contemplated to include a minimum of 200 responses by physicians, but because of this absence of a sampling frame as well as previous studies in the region to assess expected frequencies, it was not possible to do a formal power calculation. We began identifying the patients in May 2019 and ended identification of cases in March 2020, when the COVID pandemic caused closures of many hospital services. The final patients who were included were followed until their moment of death, therefore the last patients identified passed away in May 2020. The absence of a sampling frame made it impossible for the researchers to know how many eligible patients were not identified. Patients could have died in hospital (which would be known) but also outside of hospital (which would not be routinely registered by the hospitals).

### Survey instrument

The questionnaire focused on the characteristics of the end-of-life decision-making that preceded the death of the patient involved. Besides basic demographical information, the questionnaire focused on three key end-of-life decisions: a.) decisions not to initiate or to withdraw potentially life-prolonging treatment; b.) decisions to intensify measures to alleviate pain or other symptoms with hastening of death as a potential side effect; and c.) decisions to administer, supply or prescribe drugs with the explicit intention of hastening death. In case either of the first two decision types was made, we asked whether those decisions were made with the partial or full intent of hastening the death of the patient. This questionnaire was adapted from the questionnaire used in the Dutch “Death certificate study” [3, 18] (Supplementary File 1).

Physicians were further asked to choose the term that they thought best described their act: refraining from treatment, alleviation of symptoms, palliative sedation, withdrawal of futile treatment, respecting advance care directive, assisted suicide, or euthanasia.

### Ethics approval and consent, anonymity

The study protocol was approved by the research ethics committees at Pontificia Universidad Javeriana (number FM-CIE-0086-17) and NCI (Instituto Nacional de Cancerología, number INT-OFI-03581-2019). The physicians answered the questionnaire anonymously, no information on specialization, age, sex or years or experience of the participating physician was collected. Given the very sensitive nature of these decisions, which may sometimes be questionable or go against the policies of individual institutions, guaranteeing anonymity of the physicians was key to the success of this project, which implied impossibility to request informed consent (which has to be signed with identifying information). The medical ethics committees of the participating institutions studied the study protocol and allowed the researchers to omit the signed informed consent procedures which would normally apply. Neither the identified patients nor the physician signed, and no identifying information on either was registered. However, the invitation explained the objectives of the study and explicitly stated that participation was voluntary and completely anonymous. Each participating institution had a listing of the patient study codes and identifying information, kept by the research assistants, who had no access to the databases. This linking information was destroyed after the data had been collected to ensure anonymity; the researchers did not know the patients’ nor the physicians’ identifying information. All methods were performed in accordance with the relevant local and international guidelines and regulations.

### Statistical analysis

Variables with an (approximately) normal distribution were summarized as absolute frequencies and proportions, means and SD values, otherwise they were presented as medians and interquartile ranges. All analyses were done in SPSS 25.0 and R [19]. As there were multiple possible combinations of treatment decisions, we summarized combinations (intersections of multiple sets) of decisions by using UpSetR package [20]. The dataset is available as supporting information accompanying this manuscript.

## Results

We identified 348 deceased patients and obtained 261 responses from their physicians (response rate 75%; INC 86%, HUSI 61%, HUSJ 81%). The patients for whom we did not obtain a response had a similar age and sex distribution, but included more patients affiliated with the contributive insurance system. Gastric cancer was less common and breast and cervical cancer more common among patients for whom no response was obtained (Supplementary Table 1).

Of the deceased patients for whom we obtained a response from their physicians, the median time in days between death and receiving the questionnaire response was 9 days (IQ interval 6–20 days), 73% of these patients died in a hospital, 17% at home (10% missing information). Mean age of the patients was 60.2 years (SD 16.3), half of them were females and 54% were affiliated with the contributive health insurance. Most common cancer types were gastric, breast, and colorectal cancer. The distribution of patients´ general characteristics was similar between the participating hospitals, with the exception of type of health insurance and cancer types (Table 2).

**Table 2:** General characteristics of patients

	Total ***n*** = 261 (%)	HUSI ***n*** = 92 (%)	INC ***n*** = 143 (%)	HUSJ ***n*** = 26 (%)
Age^a^ (mean ± SD, median [IQR])	60.2 ± 16.3 63 [50–72]	60.8 ± 17.1 65 [47–72]	58.8 ± 15.9 60 [49–70]	66.1 ± 14.9 67 [58–76]
Sex				
Male	127 (49)	51 (55)	63 (44)	13 (50)
Female	134 (51)	41 (45)	80 (56)	13 (50)
Top 3 cancer diagnosis	Gastric 45 (18)	Gastric 15 (17)	Gastric 24 (17)	Gastric 6 (23)
	Breast 28 (11)	Colorectal 13 (15)	Breast 13 (9)	Breast 3 (11)
	Colorectal 26 (10)	Breast 12 (13)	Colorectal 12 (8)	Cervical 2 (8)
Health care insurance	Contributive 141 (54)	Contributive 89 (97)	Contributive 42 (29)	Contributive 10 (39)
	Subsidized 100 (38)	Subsidized 2 (2)	Subsidized 87 (61)	Subsidized 11 (42)
	Other & unknown 20 (8.4)	Unknown 1 (1)	Other & unknown 14 (10)	Other & unknown 5 (19)

^a^Twenty-two missing data (8.5%): HUSI = 6, INC = 10, HUSJ = 6. *HUSI* Hospital Universitario San Ignacio, *INC* Instituto Nacional de Cancerología, *HUSJ* Hospital Universitario San José

**Fig. 1:**
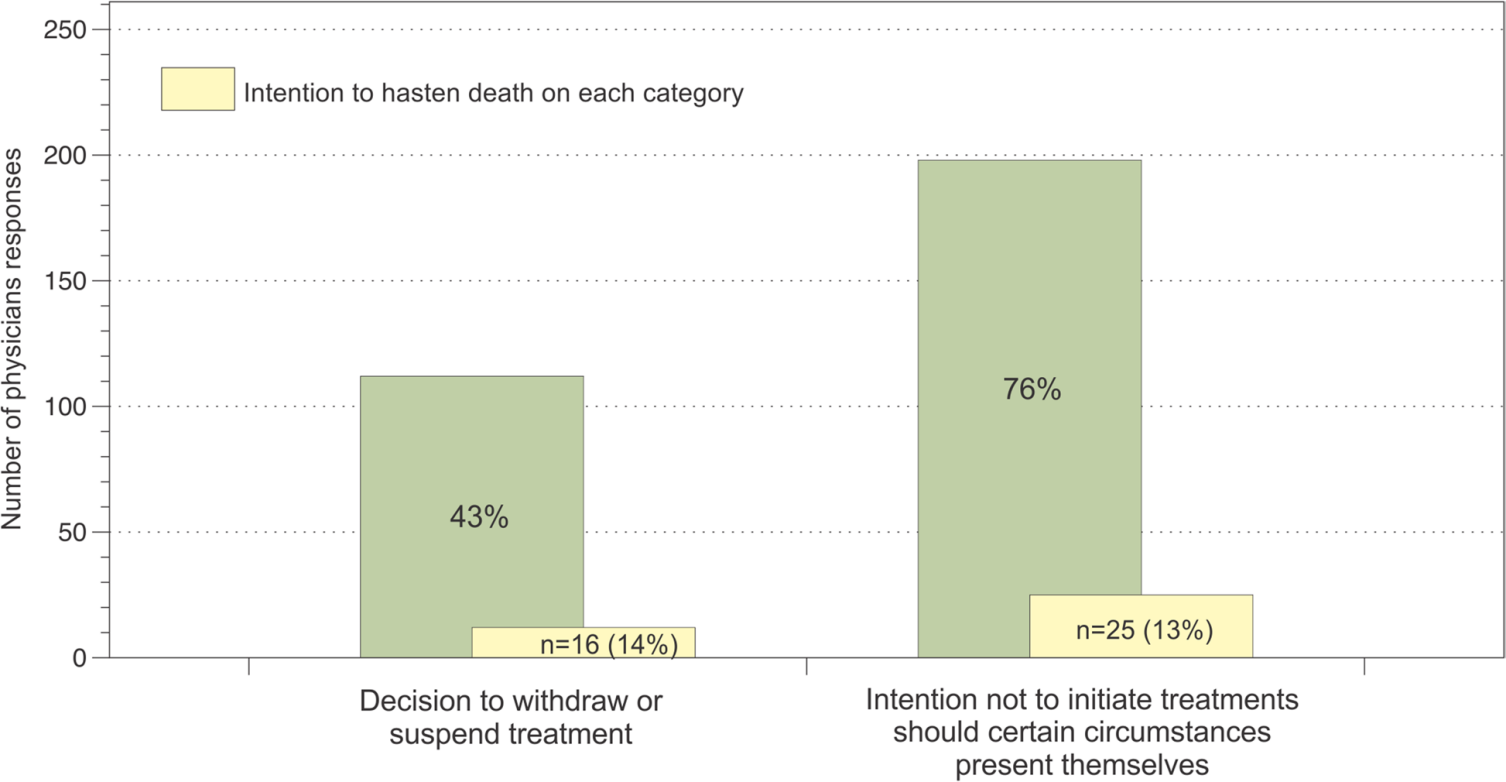
Medical decisions concerning the end of life of the patient of withdrawing or not initiating treatments (*n* = 261)

Frequencies of specific end-of-life decisions are summarized in Table 3 and combinations of decisions are depicted in Fig. 1. In almost all cases, care strategies (85%) were described by physicians as palliative care; 88% of patients received opioids (Fig. 1). In 112 patients (43%), there had been a decision to withdraw potentially life-prolonging treatment, most frequently for chemotherapy, radiotherapy or nutrition (Fig. 1). The decision to withdraw chemotherapy was often combined with the decision to suspend radiotherapy.

**Table 3:** Frequencies of requests and medical acts related to hastening the end of life as described by physicians

	Frequency (%)
Requests for active hastening of death	6 (2.2)
Granted requests for active hastening of death	2 (0.8)
Assisted suicide / suicide	0 (0)
Unknown if patient terminated own life	19 (7.2)
Medical staff administered a drug with the explicit intention to hasten the end of life of the patient without the patient’s explicit request	6 (2.2)

**Fig. 2:**
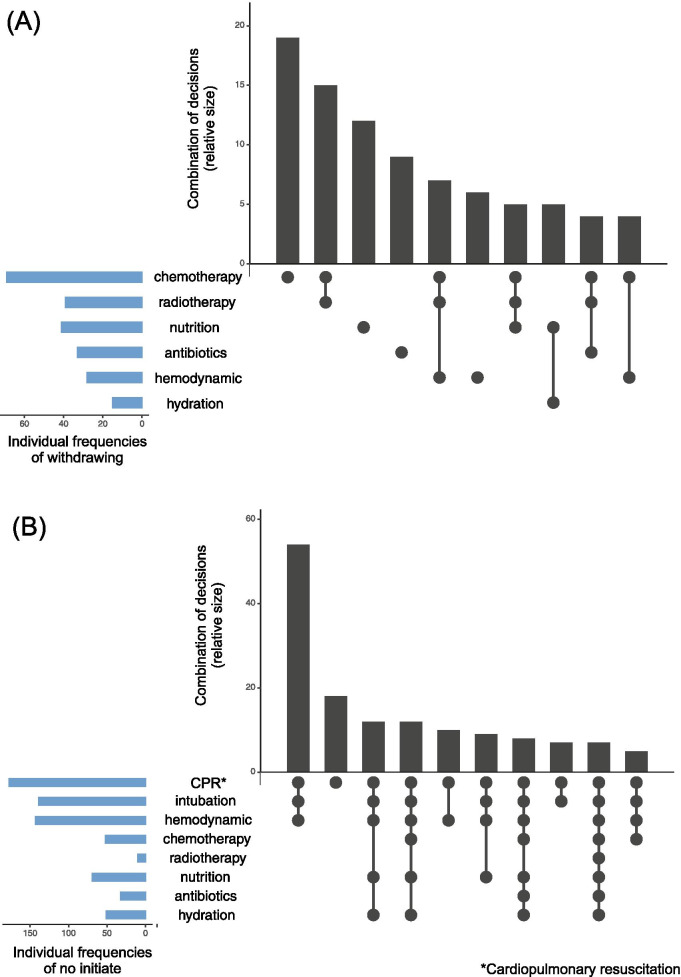
Frequencies and combinations of (**A**) withdrawing of treatment decisions, and (**B**) no initiating treatment decisions. *Physicians could select several categories of A) withdrawing of treatment decisions, or B) no initiating treatment decisions. Upset plots (above) show 1) individual frequencies of each decision (horizontal blue bars, and 2) ranking of the most frequent selected decisions, being single decisions (single points) or in combination of two or more decisions (multiple points joined by a line) [20]. For example, chemotherapy was the most selected withdrawing of treatment decision and it was made as a single choice, and the combination of CPR, intubation and hemodynamic support was the most frequent no initiating treatment decision made

Figure 2A shows the frequencies of distinct withheld treatment combinations. For the 112 patients for whom treatment withdrawal decisions were made, 16 (14%) were intended to hasten death or noted hastening death as a component in the decision-making process.

For 198 patients (76%) there had been some decision to not initiate certain treatments. Of these, not initiating resuscitation attempts and tracheal intubation were the most frequent, followed by not initiating hemodynamic support and nutrition. Not initiating resuscitation attempts was most frequently combined with not initiating intubation and not initiating hemodynamic support (Fig. 2B). There were differences among hospitals in the frequencies of decisions to not initiate treatment (HUSI 77%, INC 71%, HUSJ 96%). For 25 (13%) of the 198 patients for whom decisions not to initiate treatment were made, there was an intention to hasten death. Among these patients, decisions to not initiate resuscitation (93%) and tracheal intubation (75%), should they be needed, were frequently made. None of the physicians reported a decision to not initiate hydration among this group.

Twenty-one percent of patients were reported by physicians as having received palliative sedation and 50% of these patients received artificial hydration during this sedation period.

Six patients (2%) explicitly requested to hasten their death, and for two of them their wish was fulfilled using medication with the explicit intent to hasten the end-of-life. The medication was administered by nurses in one case and by physicians in the other case. One physician qualified this procedure as euthanasia. The reason for not fulfilling the wish to hasten death in the remaining patients was institutional rejection of applying euthanasia (four patients). Two of these patients submitted the request in another institution but passed away before finishing the procedure. One of these two patients did not have a written request.

Among 6 of the 228 patients who did *not* request to hasten death (for 27 patients we have no answer for this question), death was caused by the administration of a drug by a medical staff member with the explicit intention to hasten the end of life (or facilitate the patient to end his/her own life). For 19 patients, the physician did not know whether the patient had committed suicide or not.

In 44% (*n* = 114) of all cases, physicians indicated not to know if their patient had any advance care directives, for 25% (*n* = 66) they indicated the patient had no directive and for only 7% (*n* = 17) physicians reported the patient had an advance care directive.

Among the 145 cases where withdrawal of treatment could hasten death, 107 (74%) were not informed by their physicians regarding this potential effect of hastening death. Physicians indicated they did not speak about this potentially life-shortening effect because ‘because the treatment was clearly the best for the patient’ (38%, *n* = 55); ‘the patient was unconscious’ (37%, *n* = 53) or ‘it was not necessary to speak with the patient’ (26%, *n* = 37). In 28 of the 53 unconscious patients, physicians had a conversation with the spouse or family members of the patient, no conversations took place for the remaining 25 of these patients.

In 34% (*n* = 88) of all cases where a decision had been made, there been a conversation with family members regarding the possibility of hastening death as a result of the decision.

## Discussion

The results of this first descriptive, exploratory study regarding treatment decisions at the end-of-life of cancer patients in three Colombian hospitals showed the high frequency of not initiating treatment and withdrawal decisions. Of note, 23% of patients had palliative sedation prior to their death. According to the physicians, six patients (2%) requested explicitly to hasten death and for two of them, their wish was fulfilled.

While these figures cannot be considered representative for the situation in Colombia in general, they do provide some framework of frequency of certain decisions and of explicit requests to hasten death in this country. There are few studies documenting frequencies of distinct end-of-life decisions and even fewer specifically on cancer [21–23]. To our knowledge, no reports with observed data on euthanasia and other types of decisions from South America are available, but there are data from some European countries: of all cancer-deaths in the Netherlands in 2010, 41% of patients had received intensified measures to alleviate pain or other symptoms and 28% had received deep continuous palliative sedation [3]. In 2007 in Flanders, Belgium and the UK this proportion of palliative sedation was 15 and 17%, respectively [24]. As we did not provide a very clear definition of palliative sedation in our questionnaire, these numbers cannot be directly compared, however, our results (23% palliative sedation) are in line with these observations and it is likely that most physicians considered the term “palliative sedation” to refer to “deep palliative sedation”. In the Netherlands in 2010, of all sedated patients (including non-cancer deaths), 21% received artificial nutrition or hydration. This proportion was 2% among patients treated by family physicians, and went up to 54% among patients treated by medical specialists (54%) [18]. It seems that the frequency of applying nutrition and hydration measures (50% of sedated patients) is similar in Colombia as most of our patients were treated by medical specialists. Cultural factors and a relative absence of advance care directives among most terminal cancer patients in Colombia may influence the frequencies of specific end-of-life care decisions. Frequencies may also be somewhat biased because of the overrepresentation of hospital deaths in our study combined with the very limited availability of end-of-life care and home-based palliative care in the country [10].

In our study, non-resuscitation and non-intubation decisions were very common. However, it is hard to know whether these decisions were intentions (in case a patient would be candidate for these interventions, would we apply them?) or if the situation of non-resuscitation or non-intubation presented themselves in reality among these patients.

A recent study performed at HUSI-Colombia reports that 70% of 832 patients who died in this hospital had a do-not-resuscitate order, based on the diagnosis and prognosis of the disease. Only 3.5% of these do-not-resuscitate orders were established as advance directives, and they were obeyed in 98.3% of cases [25]. Advance care directives (AD) are formally regulated in Colombia, but their implementation suffers from several problems (see Table 4 for details) [26]. Our results show that few patients had advance directives, probably because of relatively little knowledge on the possibility of formulating ADs and lack of active information provision [27, 28]. In a comparison of physicians´ and caregivers´ knowledge regarding AD in our patient sample, there was surprisingly little knowledge and communication among both groups regarding the existence or not of ADs [29]. However, physicians often did not know whether or not patients had an AD and paternalistic attitudes, legal concerns and cultural and religious factors likely also play a role as was recently observed in a narrative review [30].

**Table 4:** The legal status of advance care directives in Colombia

The rights of persons at the end of their lives are described in the Resolución 13,437 of 1991, in Law 1737 of 2014 and the Resolución 1216 of the 20th of April 2015. Together, these describe the processes and information needed to express an advance directive (“living will”). In article 5 of the Law 1737 of 2014 the rights of patients in the last phases of their lives are described, including the right to sign advance directives, including: the right to palliative care; the right to information; to a second opinion; to actively participate in the care processes. Rights of children, adolescents and family members are also described. Article 5.4 is dedicated to the right to sign advance directives and described these as follows: “Any capable, healthy or diseased person, in full use of their legal and mental faculties, with full knowledge of the implications that this right carries may sign an advance directive. Whoever subscribes such a document indicate his/her decisions regarding undergoing unnecessary medical treatments that impede a dignified life of the patient and in the event of death the decision regarding organ donation, should this person in the future be suffering from a terminal, chronic, degenerative and irreversible disease with a strong impact on quality of life.” When providing information as part of the medical attention process, physicians are obliged to explain these rights to the patients, including the potential contents of the living will (advance care directive), explanation that the patient can revoke this will at any moment and that neither family members nor members of the medical staff can modify this will when the patient can no longer decide for himself. Medical-legal experts on the topic have summarized the following barriers to an adequate implementation of advance directives in Colombia: the scarce knowledge in the general population of the right to sign advance directives, the scarce training of healthcare professionals on the subject and the absence of national information systems or national registers of advance directives that are easy to consult by professionals to guide decision-making [23].	

Decisions not to initiate treatment may be related to medical futility, for example, deciding against fourth-line chemotherapy or antibiotic treatments in an imminently dying patient [31]. A study performed in one of the participating hospitals concluded that there had been  “disproportional treatments^1^” in 56% of cancer deaths that occurred in the hospital from 2016 to 2017, highlighting the need to establish limits on cancer treatments. Ideally, medical practice will progress to not consider death as a negative outcome, but rather focus on alleviating suffering and optimizing quality of life and death [32, 33]. Medical doctors have indicated they have difficulties deciding when to interrupt futile treatments, whereas decisions not to initiate such treatments are more easily made [32].

Decisions to withdraw or withhold “futile” or “non-beneficial” medical interventions should be discussed with the patient and relevant caregivers as problems may arise with patients persisting in their wish for “futile” treatments [16, 34]. Ideally, all treatment decisions, including non-futile interventions, will involve open and sensitive communication to ensure the patient and their caregivers are adequately informed and understand the implications of the decision. Our data indicate very low levels of communication between physicians and patients on these topics and it seems that in Colombia these decisions are mostly made by the physician (“the doctor knows best”). The six patients who were administered drugs with the explicit intent to accelerate the end-of-life but who did not explicitly request to hasten death (technically considered homicide), could be an example of this. However, these six cases could also reflect confusion among both patients and physicians regarding ADs, legal procedures for euthanasia, discussing preferences for different treatment options and other topics surrounding end-of-life care [27]. A qualitative study among healthcare professionals in Colombia showed that physicians sometimes feel an “unspoken desire” to accelerate death but do not really take this “desire” further. The same study also demonstrated the difficulty that physicians have in initiating these discussions and understanding the legal framework. Additionally, it showed that some physicians consider hastening the end of life, even without explicit patient requests, as an act of mercy [35]. This is actually in line with the 1997 court ruling on euthanasia which was based on the permissibility of compassion and solidarity with the patient, not on respect for autonomy [36]. Our study shows that very little conversation takes place between physicians, patients and family members on the dying process and end-of-life decisions, a finding in line with previous studies from countries like the United States and Denmark [37]. Possibly, these findings represent a firmly-engrained paternalistic culture which persists among Colombian health professionals. It is also likely that physicians are not well prepared to talk with their patients about death and dying.

Although euthanasia has been depenalized in Colombia for a few years (Table 1), the use of this procedure is heavily debated. Many physicians are concerned with the lack of access to palliative care services in the country, citing statements like the one from the European Association for Palliative Care, “*If euthanasia is legalized in any society, there should be special attention to avoid the underdevelopment or devaluation of palliative care and conflict between legal requirements and the personal and professional values of physicians and other healthcare professionals*” [16, 35]. In Colombia, access to palliative care services is limited and mostly concentrated in larger cities [9, 10], with many pain management specialists being considered palliative care specialists, and very limited attention paid in medical curricula to palliative care in general and end-of-life care in particular. Many healthcare institutions do not offer euthanasia, mostly for religious reasons (many hospitals are Roman Catholic institutions). Legally, they are obliged to help facilitate referral of patients to other institutions where euthanasia is performed and they should help initiate the formal process of applying for euthanasia. In reality, many patients may not have the energy and remaining life expectancy to initiate such a trajectory and additionally may not want to change institutions. Formally registered cases of euthanasia are rare: only 44 occurred in 2019 and 26 in 2020, in a population of around 49 million inhabitants [14].

There were 19 instances in our study where the physician did not know if the patient had committed suicide. We have very little further information and therefore these patients may have died and the physician just did not know the circumstances. However, considering the comment made by one oncologist in a simultaneously executed qualitative study regarding the end-of-life decision making process, there may be patients who seek accelerating their death through other routes, such as suicide (translated quote – [38]): “*No, we have not had this, but I had a patient here who committed suicide […] - died after his second chemotherapy, at home, never woke up ... we never knew the cause, but he had no complications… People don’t die overnight without having a preamble of a complication. […] I think that there are people who commit suicide and I think that many people do it because they thought about euthanasia, but people do not access easily, so they commit suicide with an overdose or something.*” Similarly, we interviewed a Colombian patient with end-stage disease mentioned having considered suicide [39]: *“Well, why am I going to live like this […], when it hurts too much. And I can’t find ... I feel those things. In fact, the last time I confessed, it was because of that […] because I had bad intentions, bad thoughts. And I wanted to do it, I don’t know why I didn’t –* Interviewer: Did you want to commit suicide? *- Aha […], but I don’t think I’m capable. I think I am not capable. It would be the easiest. But no, I don’t know if I’m capable.”*

The main limitation of the study is its sampling bias. The three participating institutions do, however, exhibit diversity in both the setting (urban versus rural and Roman Catholic versus non-Catholic) and socioeconomic classes served (private versus public hospitals with different levels of specializations offered). In order to guarantee anonymity, it was impossible to collect information regarding the specialization, age, years of experience and personal convictions of the participating physicians. It is possible that the deaths of some patients were qualified by the same physician but because of the complete anonymity and invitation procedures, this cannot be confirmed. In patients who had a physician response, the proportion that died in the hospital was higher than the national average (73% in our study versus 68.5% nationally [40]). We probably did not identify all deaths that occurred at home because the administrative system may be slower in such cases. Additionally, physicians may have been more prone to decline participation in the study for patients who did not die in the hospital as they would be less informed about those patients’ end-of-life issues. This overrepresentation of cases who died in hospitals probably increased the proportions of decisions which require hospital environments. Unfortunately, we did not have data on whether or not patients received high-intensity, invasive treatments and there is a lack of data on the character of decision-making processes (shared versus paternalistic). The high proportion of physicians who indicated not to have spoken with the patients ‘because the treatment was clearly the best for the patient’ (25%) or ‘it was not necessary to speak with the patient’ (17%) seems to indicate that the paternalistic approach is common.

The identification of potential patients for this study was terminated in the beginning of the pandemic of SARS-CoV2 – in march 2020, august 2020 had been foreseen. However, we did manage to obtain the desired minimum number of responses by physicians (> 200). The large changes that the pandemic caused in Colombian society and hospitals would probably have changed some of the “usual” habits of patients, caregivers and staff and the research team felt that closure of recruitment was indicated.

Strengths of this study include the relatively high number of deceased patients and very high participation of invited physicians in the study, although physician non-participation may not have been random and data may have been different had all physicians agreed to participate. Some “non-socially desirable” answers were obtained, even some that breach regulations - which seems to indicate that the physicians felt free to report actual events rather than distort their answers to socially desirable ones.

## Conclusions

In conclusion, all types of end-of-life decisions were being made in the three Colombian hospitals, including palliative sedation and euthanasia. Non-treatment decisions represented the majority of the end-of-life decisions made in this sample, euthanasia cases and hastening of death without a request occur – although both are relatively rare. Although the design of the study does not allow extrapolating these results to expected frequencies in the general Colombian population of cancer patients at the end of life, the results do clearly indicate the variety and circumstances of the decisions being made. There is a general lack of conversation between physicians and patients regarding wishes, advance care directives and potential life-shortening effects of treatment decisions. Since the primary aim of palliative care is to relieve suffering, it is not possible to provide palliative care without good communication with patients and their families. If physicians and other healthcare professionals do not listen to patients or lack communication skills that allow for understanding of patients’ suffering, they run the risk of inflicting further suffering through their decisions despite their best intentions [41].

## Supplementary Information

**Additional file 1.** Questionnaire: Medical end-of-life decisions.

**Additional file 2: Supplementary Table 1** Characteristics of Responders versus non-responders.

## Data Availability

The datasets used and/or analysed for the current study are available from the corresponding author on reasonable request.

## Footnotes

1 Defined in that study as invasive procedures, surgeries, complex medication, and costly interventions that began or took place during the final hospitalization period of the studied patients.
