# Supplementary material for: Medical decisions concerning the end of life for cancer patients in three Colombian hospitals – a survey study
Source: BMC Palliat Care. 2021 Oct 18;20:161. doi: 10.1186/s12904-021-00853-9 (PMC8520825; doi:10.1186/s12904-021-00853-9)
Supplement: Supplementary file 1 — Additional file 1. Questionnaire: Medical end-of-life decisions. [file 12904_2021_853_MOESM1_ESM.docx]

**Questionnaire: Medical end-of-life decisions**

1. **General information**
2. Patient code, mentioned in invitation letter: ______
3. Sex of the patient:
   1. Male
   2. Female
4. Age at the moment of death
5. Type of cancer
   1. Breast
   2. Cervix Uteri
   3. Lung
   4. Prostate
   5. Stomach
   6. Colon and rectum
   7. Others (specify):
6. Type of affiliation to Social Security System (SGSSS):
7. Contributive
8. Subsidized
9. Special/exceptional regime
10. Not affiliated
11. I don´t know
12. The information that I have with regards to the circumstances of death of the patient is:
    1. Having known the patient prior to or around the moment of death
    2. Contact with healthcare workers or third persons after the moment of death of the patient
13. Was the death sudden and unexpected?
    1. Yes
    2. No
14. **Medical decisions made around the moment of death of the patient**
15. Did you decide to withdraw a treatment from this patient, or do know have knowledge that another physician did so? Please mark all answer options that apply
    *In this study “treatment” includes artificial nutrition and hydration
16. Yes, nutrition was suspended
17. Yes, hydration was suspended
18. Yes, chemotherapy suspended
19. Yes, radiotherapy suspended
20. Yes, antibiotics treatment suspended
21. Yes, hemodynamic therapies suspended (vasopressors, inotropic drugs, etc.)
22. No treatments were suspended or withdrawn
23. Other treatment were suspended/withdrawn, please mention which:
24. Was accelerating the end of life part of this withdrawal decision?
25. Yes
26. No
27. Did you or another physician decide to not initiate certain treatments for this patient? Please mark all answer options that apply.
    *In this study “treatment” includes artificial nutrition and hydration
28. Chemotherapy was not initiated
29. Radiotherapy was not initiated
30. Nutrition was not initiated
31. Hydration was not initiated
32. Antibiotics treatment was not initiated
33. Orotraqueal intubation was not initiated
34. Advances reanimation manoeuvres were not initiated
35. Hemodynamic Support was not initiated
36. All treatments were initiated
37. Some treatments were initiated
38. Other treatments were not initiated, please mention which:
39. Was accelerating the end of life part of this not initiating decision?
40. Yes
41. No
42. Did you or another physician decide to intensify pain or other symptom management for this patient?
43. Yes, the patient received medication to alleviate pain and other symptoms
44. No, no medication was administered for such purposes
45. Was accelerating the end of life part of this intensification of symptoms treatment decision?
46. Yes
47. No
48. Was the death the consequence of one or more of the following decisions, that you or another physician made with the explicit intention to not prolong suffering?

*In this study “treatment” includes artificial nutrition and hydration

1. Not initiating treatment
2. Suspending or withdrawing a treatment
3. Administration of medication for symptom control
4. The death of the patient was not a consequence of the medical interventions
5. Was the death caused by the use of a prescribed drug or a drug administered by someone of the medical staff with the explicit intention to accelerate the end of life (or facilitate the patient to end his/her own life)?
6. Yes
7. No
8. Did you or another physician make any of the following decisions in this patient?
9. Not initiating treatment
10. Suspending or withdrawing a treatment
11. Pain – symptom management
12. None of these decisions were made

**III- Participation of family members or other caregivers**

1. Was there a conversation with the family members or other caregivers regarding the possibility of accelerating the end of life as a result of the intervention or non-intervention referred to in the previous section?
2. Yes, with the partner/family of the patient
3. Yes, with others
4. No

#### Was the decision regarding this intervention taken as a result of an explicit request from the family members of the patient?

#### Yes

#### No

1. Was there a conversation with the patient regarding the possibility of accelerating the end of life as a result of the intervention or non-intervention referred to in the previous section?
2. Yes, at the moment the decision was taken
3. Yes, some time before implementing the decision
4. There was no conversation regarding this topic

#### Who initiated this conversation?

1. The patient
2. You or another physician
3. Nursing or other care-providing staff
4. The partner or family member of the patient
5. Another person: (specify)

#### Patients´ wishes

#### Did you consider the patient to be able to understand his or her medical condition and to take health-related decisions adequately at the moment of the conversation?

#### Yes, completely

#### Yes, partially

#### No, the patient was not able to

1. Why was there no conversation regarding the possibility to accelerate the end of life as a result of the medical decision with the patient?
2. The medical treatment provided was clearly the best for the patient
3. The dialogue would have caused more harm than benefit to the patient
4. The patient was too young or too old
5. The patient was unconscious
6. The patient had a mental problem or dementia which made a dialogue impossible
7. It was not necessary to talk to the patient regarding this decision
8. Other, please specify:

#### To your best knowledge, had the patient ever expressed a desire to accelerate the end of life?

#### Yes, in a very clear way

#### Yes, but not explicitly

#### The patient had not expressed such a wish

#### To your best knowledge, did the patient have any advance directives?

#### Yes, the patient had a written directive

#### Yes, the patient had a verbal directive

#### Yes, the patient had both a written and verbal directive

#### The patient had no directives to my knowledge

#### I don´t know

#### At the moment the patient had made his request, was the patient considered to be capable of evaluating his/her medical condition and make decisions regarding his/her treatment?

#### Yes, completely

#### Yes, partially

#### The patient was not able to

#### Was the medical decision referred to in the previous section taken because of an explicit directive (either verbal or written) by the patient?

#### Yes, the patient had requested it

#### The patient had not requested this treatment

#### Although the patient had a directive (verbal or written), the request was not fulfilled.

1. **Regarding accelerating the end of life**

#### Did you speak to other healthcare professionals regarding the possibility of hastening death of the patient before making the treatment or non-treatment decision (referred to in the previous section)? Please mark all answer options that apply

#### Yes, in a multidisciplinary team

#### Yes, with one or more physicians

#### Yes, with nursing staff

#### No

#### What was the objective of your conversation with other healthcare professionals about the possibility of hastening death of this patient?

#### Decide in multidisciplinary team

#### Exchange information and opinions with colleagues

#### Formal consultation in the framework of legal requirements for euthanasia (for example, application of the rules set out in Resolution 1216 of 2015)

#### To feel more certain of the decision

#### LIKERT-scale questions:

#### What were the main reasons to decide on applying a treatment with a potentially life-shortening effect of your patient? Please assign scores to the options, 1 being the most important, 10 the least important reason.

#### Pain (severe)

#### Other severe symptoms

#### Loss of dignity

#### Patient´s wish

#### Family´s wish

#### Compassion

#### Foreseen suffering because of the patient´s disease

#### No perspective of improvement

#### Continuation of treatment would have been too difficult for the patient

#### There was no treatment that somehow could have improved quality of life of the patient

#### Other, please specify

#### How would you describe the medical intervention that you refer to in this questionnaire?

#### Symptom control

#### Non-treatment decision

#### Euthanasia

#### Physician-assisted suicide

#### Palliative sedation

#### Suspension of futile treatments

#### Respecting advance directives

#### Other, please specify

31. Was the patient hospitalized during the last 30 days of his life?

#### Yes, and he died hospitalized

#### Yes, but he did not die hospitalized

#### The patient was not hospitalized

#### What was the treatment objective during the last few days of patient´s life?

#### Cure

#### Palliation

#### Other, please specify

#### In general, during the last 30 days of this patients´ life, which professionals were involved in the care for the patient? Please select all that apply.

#### Oncologists

#### Palliative medicine specialists

#### Specialists in pain management

#### Psychiatrist or psychologist

#### Other medical specialists

#### Home-care physician

#### Nutritionist

#### Respiratory therapists / physiotherapist

#### Nurse

#### Priest, pastor or other religious representant

#### Other, please specify

34. Was the patient diagnosed with dementia or delirium?

1. Yes
2. No

#### Did the patient receive palliative sedation?

1. Yes
2. No
3. During the period of palliative sedation, was artificial nutrition or hydration administered to the patient?
4. Yes
5. No
6. Did the patient receive morphine or derived drugs during the last 24 hours before death?
7. Yes
8. No

38. Was a higher dose of morphine (or derived drugs) than needed for symptom control and other symptoms applied to the patient?

1. Yes
2. No

39. Was there a pre-specified agreement on not performing CPR manoeuvres should the patient enter in a cardiorespiratory arrest?

1. Yes, together with the patient
2. Yes, together the patient and relatives
3. Yes, with other caretakers of the patient
4. No, but based on the clinical conditions and prognosis of the patient, no reanimation was indicated
5. Nothing was agreed upon
6. I don´t know

#### 40. Had the patient explicitly requested help in accelerating the end of life?

1. Yes
2. No

41. Was this request fulfilled?

1. Yes
2. No

42. If the patient had explicitly requested acceleration of the end of life, why was this wish not fulfilled?

1. Patient passed away before being able to respect the wish
2. Patient´s suffering did not comply with legal requirements described in the resolution
3. There was no well-established advance directive from the patient
4. The patients request was not made voluntarily
5. Because of personal objections
6. In our institution these procedures are not performed
7. Patient withdrew the request
8. Other (specify)

#### 43. Was euthanasia applied to this patient?

1. Yes
2. No
3. If the previous question was answered affirmatively, who administered the drug?

45. Do you know if the patient ended his own life without medical assistance (did patient commit suicide)?

- 1. Yes, the patient ended his own life
  2. The patient did not end his own life
  3. I am not sure

#### 45. Would you say this patient died peacefully?

1. Very peacefully
2. Moderately peacefully
3. Restless/uneasy
4. Very restless

#### 46. As a physician, do you feel at ease with the medical accompaniment during the process of dying of this patient?

1. Yes, I am comfortable with the provided care/assistance
2. I think some interventions could have been different
3. No, I think we could have managed this in a better way

47.  Who was the principal accompaniment of the patient during the process of death?

1. The patient’s partner or family members
2. Medical staff
3. The patient was alone
4. I don´t know

48. Are/were you worried for you or your colleagues regarding the medical end-of-life decisions taken for this patient for ethical, legal or administrative reasons?

1. Yes
2. No

49. Do you think this type of decisions should be taken:

1. In the private atmosphere of the patients, their family, and physicians
2. With the help and approval of the healthcare institution to which the patient and doctor pertain
3. Other, specify
