# Supplementary material for: Medical decisions concerning the end of life for cancer patients in three Colombian hospitals – a survey study
Source: BMC Palliat Care. 2021 Oct 18;20:161. doi: 10.1186/s12904-021-00853-9 (PMC8520825; doi:10.1186/s12904-021-00853-9)
Supplement: Supplementary file 2 — Additional file 2: Supplementary Table 1 Characteristics of Responders versus non-responders. [file 12904_2021_853_MOESM2_ESM.docx]

Supplementary Table 1: responders versus non-responders

|  | **Responders** | **Non-responders** |
| --- | --- | --- |
|  | **N=261** | **N=88** |
| **Mean age (SD)** | 60.8 (16.1) | 61.5 (14.8) |
|  |  |  |
| **Sex** | **% (N)** | **% (N)** |
| **Males** | 49.0 (128) | 37.5 (33) |
| **Females** | 51.0 (133) | 62.5 (55) |
| **Type of health insurance** |  |  |
| **Contributive** | 53.6 (140) | 77.3 (68) |
| **Subsidized** | 73.3 (118) | 21.6 (19) |
| **Other** | 1.0 (3) | 1.0 (1) |
| **Cancer type** |  |  |
| **Gastric** | 17.6 (46) | 9.1 (8) |
| **Colorectal** | 9.6 (25) | 10.2 (9) |
| **Breast** | 10.0 (26) | 12.5 (11) |
| **Lung** | 5.4 (14) | 6.8 (6) |
| **Cervix uteri** | 4.6 (12) | 11.3 (10) |
| **Hematological cancer** | 7.3 (19) | 6.8 (6) |
